# Effectiveness of acceptance and commitment therapy for distress, emotion regulation, and self-compassion in patients with cardiovascular disease: a randomized clinical trial

**DOI:** 10.47626/2237-6089-2023-0685

**Published:** 2025-03-27

**Authors:** Arash Fattahi, Fatemeh Mazini, Nasrin Jaberghaderi, Fatemeh Rajabi, Mehdi Derakhshani, Mohammad Laki

**Affiliations:** 1 Islamic Azad University Department of Psychology, Kermanshah Branch Kermanshah Iran Department of Psychology, Kermanshah Branch, Islamic Azad University, Kermanshah, Iran.; 2 Islamic Azad University Department of Psychology, Sari Branch Sari Iran Department of Psychology, Sari Branch, Islamic Azad University, Sari, Iran.; 3 Kermanshah University of Medical Sciences Faculty of Medicine Department of Clinical Psychology Kermanshah Iran Department of Clinical Psychology, Faculty of Medicine, Kermanshah University of Medical Sciences, Kermanshah, Iran.; 4 Shahid Beheshty University School of Psychology and Educational Sciences Department of Psychology Tehran Iran Department of Psychology, School of Psychology and Educational Sciences, Shahid Beheshty University, Tehran, Iran.; 5 Mashhad University of Medical Sciences Faculty of Medicine Department of Clinical Psychology Mashhad Iran Department of Clinical Psychology, Faculty of Medicine, Mashhad University of Medical Sciences, Mashhad, Iran.; 6 Islamic Azad University Department of Psychology, Andimeshk Branch Andimeshk Iran Department of Psychology, Andimeshk Branch, Islamic Azad University, Andimeshk, Iran.

**Keywords:** Acceptance and commitment therapy, distress, emotion regulation, self-compassion, cardiovascular

## Abstract

**Objective:**

Cardiovascular patients experience various psychological problems due to the conditions caused by their disease, making it worse if left untreated. The purpose of the current study was to evaluate the effects of acceptance and commitment therapy (ACT) on distress, emotion regulation, and self-compassion in patients with cardiovascular disease.

**Methods:**

This study was a randomized clinical trial with pre-test, post-test, and 2-month and 4-month follow-up periods accompanying a control group and an experimental group. Patients filled out questionnaires at four stages; a Depression, Anxiety, and Stress Scale (DASS-21), an Emotion Regulation Questionnaire (ERQ), and a Self-Compassion Scale (SCS). The experimental group underwent a treatment protocol based on ACT. Data were then analyzed using SPSS-25 with repeated measures analysis of variance (ANOVA).

**Results:**

ACT significantly reduced depression, anxiety, and stress, enhanced self-compassion, and improved emotion regulation in cardiac patients. Between-subjects (group) partial Eta squared (η²) for depression, anxiety, stress, reappraisal, suppression, and self-compassion were 0.61, 0.64, 0.66, 0.62, 0.66, and 0.65, respectively. Treatment efficacy was maintained at the 2-month and 6-month follow-up visits.

**Conclusion:**

The results of this study suggest that treating cardiac patients’ psychological problems in an ACT-focused manner may have an impact on how well they respond to their treatment.

## Introduction

Cardiovascular disease (CVD) is the most common noncontagious disease and refers to a group of diseases that affect blood vessels or the heart.^1^ According to the World Health Organization (WHO), CVD is one of the leading causes of death, killing 17.9 million people in 2019.^2^ Coping with a serious illness such as CVD can lead to increased levels of fear, personal health concerns, and psychological distress and to changes in health-related quality of life.^3^ CVD has both economic and psychological consequences. For example, affected individuals experience depression, anxiety, and mental distress which can disrupt their recovery process.^4^ The disease can also disrupt emotion regulation and lead to poor self-compassion in sufferers.^5^ Individuals who lack the emotional and/or mental capacity to effectively handle stressful situations may be more prone to psychiatric pathologies.^6,7^ Accordingly, anxiety, depression, or any perceived psychosocial stress these patients must deal with are associated with rapid disease progression, increased future cardiac events, decreased quality of life, increased health care costs, and poor long-term psychological adjustment, all of which are associated with poorer prognosis.^7,8^ There is substantial evidence from epidemiological, psychological, psychiatric, cardiological, and public health studies supporting these associations.^9^

Studies investigating cardiovascular risk factors suggest that these risk factors reduce cortical blood flow, cause white matter damage, and cause dysfunction in frontal-subcortical circuits that regulate emotions.^10,11^ Emotion regulation involves experiencing, processing, and modifying emotional responses. Consequently, when a range of emotions are evoked as a result of a health event, the inability to effectively manage those emotions can limit self-care activities and affect mental and physical health.^11^ A study by Jentsch and Wolf^12^ examined the function of controlling negative emotions in enhancing psycho-physiological reactions to an acute psychosocial stressor. Their study's findings demonstrated that adaptive patterns and overall adaptability of cardiovascular, neuroendocrine, and psychological responses are fostered by cognitive reappraisal (rather than expressive suppression).

Although the relationship between emotion regulation styles and well-being is well-documented, there is limited research on how these strategies affect physical health. Another factor that may be related to emotion regulation and coping styles in these patients is self-compassion.^13^ Self-compassion is about being kind to yourself rather than being judgmental, knowing that we are the same as others when dealing with isolation, and thereby being aware of one's pain and problems rather than ignoring them.^14^ With this attitude, we can make positive changes. Also, with self-compassion, rather than avoiding unpleasant experiences, we strive to be kind and tolerant of unpleasant emotions. High self-compassion is associated with willingness to engage in self-care/health-promoting behaviors, reduced disease risk, and improved physical health. In contrast, low self-compassion is associated with unhealthy behaviors (such as smoking and excessive alcohol consumption), increased risk of disease, and shortened lifespan.^15^ Therefore, there is an urgent need to pay special attention to this factor in cardiovascular patients.

Cardiac rehabilitation (CR) after a cardiac event is recommended in such patients and is usually delivered in groups. CR includes exercise classes, training, and stress management techniques to improve CVD risk profile, physical fitness, and psychological function.^16,17^ This includes counseling, meditation, and cognitively challenging negative thinking, although its psychological component is non-standard. Related systematic reviews and meta-analyses also showed mild to moderate effects of CR on symptoms of anxiety and depression.^18^ On the other hand, the quality of evidence supporting psychological treatments for anxiety and depression in CVD is generally low. Therefore, treatment of these disorders should use third-wave cognitive-behavioral therapy, which focuses on concepts such as mindfulness, acceptance, values, and goals.^19^ These therapies address the areas in which cognitive behavioral therapy falls short. The relational framework theory (RFT) provides a theoretical foundation for the primary processes involved in psychopathology and dysfunctional emotional regulation and is the foundation for acceptance and commitment therapy (ACT), one of the third wave therapies.^20^ ACT promotes acceptance and tolerance of inner experiences through use of strategies such as present-moment awareness, cognitive flexibility, and commitment to values.^21^ Increasing psychological flexibility is the major treatment objective of ACT. Instead of avoiding and suppressing mental occurrences, ACT techniques assist people in learning to embrace and observe them.^22^ As shown earlier, developing psychological flexibility allows people to change their relationship to their inner experience (rather than changing the experience itself), and to change the desired behavior (e.g., more physical activity) even in the face of difficult thoughts.^23^

Effective treatment of anxiety, depression, and mental health problems in patients with CVD should be a focus when establishing chronic patient care programs, and it also needs to be a priority for policy makers and clinical trials. Therefore, this study aimed to conduct a clinical trial to examine the efficacy of ACT for psychopathology, emotion regulation, and self-compassion in patients with cardiovascular diseases from Kermanshah, Iran.

## Methods

### Ethical considerations

This study was a randomized clinical trial with experimental and control groups. The study was approved by the ethics committee of Kermanshah University of Medical Sciences (IR.KUMS.REC.1399.033).

### Procedure

The sample was selected using a convenience sampling method. Patients were selected after advertisements were distributed to hospitals in Kermanshah. After attending a clinical interview and answering the questionnaires, participants who met the criteria for emotional problems were included in the study. The study consisted of two groups. Patients in the control group received only cardiovascular drugs. In contrast, the experimental group members also received ACT treatment in addition to CVD treatment. Treatment was given once a week in eight 90-minute sessions. Participants were evaluated four times: pre-test, post-test, 2-month follow-up, and 4-month follow-up. Measurements were taken 2 days before the intervention, 2 days after the intervention, 2 months after the end of the treatment, and 4 months after the end of the treatment. The treatment was administered by a faculty member who had the necessary information about the treatment program. ACT was performed based on the Eifert and Forsyth treatment protocol.^24^ The treatment consisted of eight sessions. The contents of the sessions are shown in Table 1.

**Table 1:** Summary of the acceptance and commitment therapy (ACT) protocol

Module	Content
1	Introduction, therapeutic relationship, informing participants about the subject of the research, treatment goals and therapist agreements, an introduction to ACT, and defining the rules governing the sessions
2	Discussing experiences and evaluations, function as a measure, developing creative hopelessness, practice accepting thoughts and feelings, life enhancing exercises, assessing a person's willingness to change, summarizing the material discussed at the session, and setting the assignment
3	Introducing control as a problem, introducing willingness as another possible response, engaging in purposeful actions, choosing valuable directions, value-based behavior as a substitute for anxiety control, discussing problems and challenges in acceptance of the disease, investigating exercises for the next session, and setting the assignment
4	Application of cognitive defusion techniques, disorganizing problematic chains of language, weakening the fusion of self with thoughts and emotions, learning to accept anxiety by mindfulness, internal control versus external control, and intervention in the functioning of problematic chains of language and metaphors
5	Observing self as context, weakening conceptualized self and strengthening observer self, self as content versus self as context, and playing volleyball with anxious thoughts and feelings
6	Application of mindfulness techniques, models of "mind" as a separate entity, learning to see experiences as process, practicing emotions and experiences to enrich life, identifying patients’ life values and focusing on these values, summarizing the material discussed at the session, investigating exercises for next session, and setting the assignment
7	Introducing the concept of values, pointing out the disadvantages of focusing on results, discovering the practical values of life, describing differences between values, goals, and common mistakes, identifying the internal and external barriers
8	Understanding the nature of acceptance and commitment, determining patterns of actions aligned with values, identifying value driven behavior plans and creating a commitment to them

### Participants

This study included 53 participants. Sample size was obtained either from previous studies or calculated using the following formula.^25^


$$\begin{array}{l} n=\frac{{\left(Z_{1-\alpha /2}+Z_{1-\beta }\right)}^{2}\left(s_{1}{}^{2}+s_{1}{}^{2}\right)}{{\left(\mu_{1}-\mu_{2}\right)}^{2}}\\ n=\frac{\left(10.49\right)\left(4.87^{2}+2.66^{2}\right)}{{\left(6.03-2.93\right)}^{2}}=21.71 \end{array}$$

**Figure 1:**
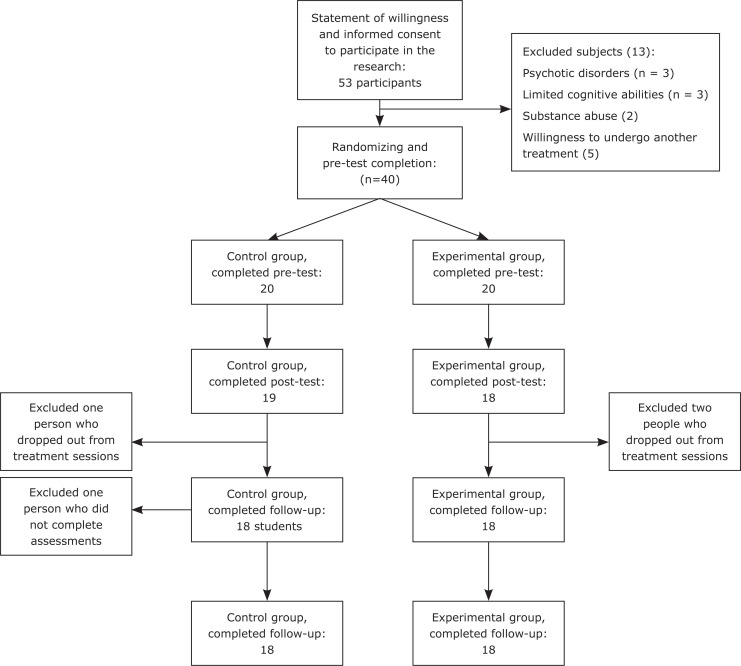
Participants’ diagram in pre-test, post-test and follow-up phases

Considering probable losses from samples, the result was increased to 40 (Figure 1).

After screening, 20 participants were selected for the experimental group and 20 were assigned to the control group. During the research process, four participants were excluded for specific reasons; one of the participants in the experimental group and two participants in the control group did not complete the sessions, so they did not complete the post-test or the follow-up phases. In addition, one participant in the experimental group did not complete the follow-up period due to recurrence of CVD symptoms. The average monthly income of the participants was 300 dollars. Statistical analysis of the demographic characteristics of the participants revealed no significant differences between the subjects with respect to demographic characteristics.

After determining the sample size, all patients, both male and female, were evaluated using the Anxiety Disorders Interview Schedule for the Diagnostic and Statistical Manual of Mental Disorders, 4th edition (ADIS-IV) to confirm initial patient eligibility for the study. The study included cardiovascular patients who had emotional problems according to clinical interviews and questionnaire cutoff scores for depression, stress, and anxiety. All the interviews and questionnaires were conducted by a psychiatrist. Inclusion criteria for this study were as follows: 1) diagnosis of CVD based on specialist diagnosis; 2) at least 18 years of age and with at least a high school diploma; 3) agreeing to participate in the study; 4) no history of substance abuse for at least 1 year. Patients who met the following criteria were excluded from the study: 1) inability to read or understand Persian sufficiently to fill out the questionnaires and treatment plan; 2) history or current presentation of disorders such as schizophrenia, bipolar disorder type I or II that destroyed realism; 3) suicidal-homicidal ideation; 4) presence of acute physical problems; 5) substance abuse; 6) concomitant participation in other psychological interventions; 7) failure to attend two or more treatment sessions. Participants completed an informed consent form prior to the procedure. Also, they were completely free to leave the treatment sessions whenever they wished. All information regarding treatment sessions will be treated as absolutely confidential. Participants who wish to be informed about research results will be provided with the relevant information. Participants were then randomly assigned to experimental and control groups. Treatment sessions were conducted by the researcher at the hospital in the form of eight 90-minute sessions, in a group setting, based on the Eifert and Forsyth treatment protocol.^24^ The psychiatrist had been appropriately educated about the questionnaires. All follow-up examinations were performed by the designated person to avoid bias.

### Instruments

#### Depression, Anxiety, Stress Scale (DASS-21)

This questionnaire consists of 21 questions rated on a Likert scale. The results of the factor analysis of this scale showed that 68% of the total variance of the scale is measured by these three factors. Its convergent validity for stress, anxiety, and depression were 0.62, 0.57, and 0.77, respectively. Cronbach's alpha coefficients for stress, depression, and anxiety have been reported as 0.97, 0.92, and 0.95, respectively.^26^ The validity and reliability of this questionnaire has been examined in Iranian students, and the results yielded test-retest reliability of 0.80, 0.76, and 0.77 for depression, anxiety, and stress, respectively. Cronbach's alpha values for the depression, anxiety, and stress scales were 0.81, 0.74, and 0.78, respectively. Its convergent validity for stress, anxiety, and depression was 0.59, 0.57, and 0.70, respectively.^27^

#### Self-Compassion Scale (SCS)

This scale comprises 26 items with a five-point Likert scale that measures three bipolar components in the form of six subscales. These sub-scales are self-compassion, self-judgment, mindfulness, over-identification, common humanity, and isolation. The Cronbach's alpha coefficient of 0.92 indicates a high level of internal consistency for the original version of this scale. Satisfactory convergence and divergence validity and test-retest reliability have also been reported on this scale. Its divergent validity was −0.51, which is good validity.^28^ The six-factor structure of the questionnaire was confirmed in a sample of Iranian students with a Cronbach's alpha coefficient of 0.86 for the whole scale. Its divergent and convergent validity were −0.36 and 0.26, which is significant. Cronbach's alpha coefficients for the subscales were also in the range of 0.79 to 0.85.^29^

#### Emotion Regulation Questionnaire (ERQ)

This questionnaire was developed by Gross and John in 2003. It consists of 10 items that assess respondents’ tendency to regulate their emotions through two main strategies: cognitive reappraisal and expressive suppression. Cronbach's alpha coefficients for suppression and reappraisal were 0.73 and 0.79, respectively. Its test-retest reliability was 0.69 for both suppression and reappraisal.^30^ Subjects respond to each item using a 7-point Likert scale ranging from 1 (strongly disagree) to 7 (strongly agree). Its validity and reliability have been investigated in an appropriate Iranian sample. Its convergent validity and divergent validity for suppression and reappraisal were 0.28 and −0.24, respectively. Its Cronbach's alpha coefficients were 0.76 for the cognitive reappraisal sub-scale and 0.72 for suppression.^31^

## Results

A total of 40 individuals participated in this study. Their demographic characteristics are presented in Table 2. The results of the analysis showed that there were no significant differences between the two groups.

**Table 2:** Demographic characteristics of the subjects

Parameters		Experimental group	Control group	p-value
Grade				0.24
	Diploma	8 (0.40)	9 (0.45)	
	Bachelor's	9 (0.45)	7 (0.35)	
	MSc	2 (0.10)	3 (0.15)	
	PhD	1 (0.5)	1 (0.5)	
				
Marital status				0.52
	Single	4 (0.20)	3 (0.15)	
	Married	16 (0.80)	17 (0.85)	
				
Age, years		45.50 ± 0.96	46.06 ± 1.09	0.36
				
Gender				0.41
	Male	12 (60.00)	13 (65.00)	
	Female	8 (40.00)	7 (35.00)	
				
Occupational status				0.27
	Employed	16 (85.00)	15 (75.00)	
	Unemployed	4 (15.00)	5 (25.00)	

Before conducting the analysis of variance (ANOVA) with repeated measures, and in order to test compliance with the assumptions, the results of Box's M test, Mauchly's sphericity test, and Levin tests were assessed. Since Box's M test was not significant for depression (Box's M = 17.25, p = 0.13), anxiety (Box's M = 14.78, p = 0.23), stress (Box's M = 16.74, p = 0.14), self-compassion (Box's M = 12.53, p = 0.36), reappraisal (Box's M = 12.34, p = 0.37), or suppression (Box's M = 14.66, p = 0.23), the assumption of homogeneity of the variance-covariance matrices was met. Also, the non-significant Levene's test results for depression (Levene = 1.005, p = 0.32), anxiety (Levene = 0.17, p = 0.68), stress (Levene = 0.07, p = 0.78), self-compassion (Levene = 0.06, p = 0.79), reappraisal (Levene = 0.25, p = 0.61), and suppression (Levene = 1.24, p = 0.27) show that the assumption of equality of variances between groups was met and the error variance of the dependent variables were equal in all groups. Finally, examination of the results of Mauchly's sphericity test showed that this test was not significant for any of the variables and, therefore, the assumption of equality of variances within subjects was accepted.

Table 3 presents the means and standard deviations of the dependent variables for the four phases pre-test, post-test, 2-month follow-up, and 4-month follow-up.

**Table 3:** Mean and standard deviation of the variables

Variable/Group		Pre-test	Post-test	2-month follow-up	6-month follow-up
Depression					
	Experimental	12.94 (1.79)	9.11 (1.32)	9.27 (1.41)	10.33 (2.50)
	Control	13.55 (1.29)	13.22 (1.83)	13.55 (1.58)	13.77 (1.59)
					
Anxiety					
	Experimental	15.05 (1.16)	11.44 (1.61)	11.66 (1.14)	12.33 (1.49)
	Control	14.16 (1.24)	15.83 (0.98)	15.50 (1.29)	14.94 (1.34)
					
Stress					
	Experimental	19.27 (1.80)	15.72 (1.63)	16.38 (1.53)	16.94 (1.35)
	Control	20.88 (2.02)	21.83 (2.74)	22.05 (2.41)	22.66 (2.47)
					
Self-compassion					
	Experimental	81.72 (0.79)	89.38 (3.56)	89.07 (2.57)	88.07 (2.85)
	Control	79.05 (0.93)	83.33 (1.84)	82.83 (2.22)	82.33 (2.58)
					
Suppression					
	Experimental	22.16 (1.33)	16.27 (1.22)	17.00 (1.18)	17.88 (1.45)
	Control	20.55 (1.75)	21.22 (0.73)	21.50 (1.61)	22.16 (1.79)
					
Reappraisal					
	Experimental	20.05 (1.16)	24.88 (1.23)	23.66 (1.53)	22.77 (1.43)
	Control	18.88 (1.83)	20.83 (1.79)	20.33 (1.90)	19.77 (2.05)

Table 4 indicates that ACT significantly changed the variables of depression, stress, anxiety, self-compassion, and emotion regulation. These changes were significant and persistent over time. As the table shows, the highest effect size was for stress, followed by suppression, self-compassion, anxiety, reappraisal, and depression, respectively.

**Table 4:** Mixed analysis of variance (ANOVA) with repeated measures

Variable/Source		SS	Df	MS	F	Sig.	Eta squared (η²)
Depression							
	Interaction (Time*Group)	30.42	1	30.42	10.679	0.01	0.23
	Within-subjects (Time)	85.944	3	28.648	15.45	0.01	0.31
	Between subjects (Group)	324.00	1	324.00	53.89	0.01	0.61
							
Anxiety							
	Interaction (Time*Group)	45.00	1	45.00	36.08	0.01	0.51
	Within-subjects (Time)	27.167	2	9.056	8.56	0.01	0.20
	Between subjects (Group)	225.00	1	225.00	62.591	0.01	0.64
							
Stress							
	Interaction (Time*Group)	63.606	1	63.606	21.60	0.01	0.50
	Within-subjects (Time)	37.056	2	12.352	7.68	0.01	0.18
	Between subjects (Group)	821.778	1	821.778	68.212	0.01	0.66
							
Reappraisal							
	Interaction (Time*Group)	11.001	1	11.001	4.42	0.04	0.11
	Within-subjects (Time)	224.410	2	74.803	50.238	0.01	0.59
	Between subjects (Group)	303.495	1	303.340	55.495	0.01	0.62
							
Suppression							
	Interaction (Time*Group)	133.472	1	133.472	45.16	0.01	0.57
	Within-subjects (Time)	139.861	2	46.620	30.515	0.01	0.47
	Between subjects (Group)	330.028	1	330.028	67.348	0.01	0.66
							
Self-compassion							
	Interaction (Time*Group)	39.200	1	39.200	10.87	0.01	0.24
	Within-subjects (Time)	825.500	1	275.167	111.23	0.01	0.76
	Between subjects (Group)	961.00	1	961.00	65.757	0.01	0.65

Table 5 reports the results of pairwise comparisons. As the table shows, there were significant differences between control and experimental groups.

**Table 5:** Pairwise comparisons (experimental vs. control group)

Variable	Mean difference	p-value
Depression	-2.95	0.01
Anxiety	-2.50	0.01
Stress	-4.77	0.01
Reappraisal	2.90	0.01
Suppression	-3.02	0.01
Self-compassion	5.16	0.01

## Discussion

The present study was conducted with the aim of investigating the effectiveness of ACT for reducing psychopathology and improving emotion regulation and self-compassion in cardiovascular patients. The results indicate that a therapeutic period of ACT can have long-term effects. The findings of this study are consistent with research into and meta-analyses of ACT in the field of treating mental health problems in patients with chronic pain and social anxiety.^23,32–34^

This study's early findings showed that ACT is effective for reducing psychological problems such as depression, anxiety, and stress in cardiovascular patients. This finding is consistent with the results of a systematic meta-analysis by Lee et al.,^26^ which reported that ACT has moderate to high efficacy for reducing anxiety, depression, and stress as well as for increasing hope in patients with cancer.^35^ Regarding ACT, acceptance-based interventions require clients to focus on thinking about meaningful living rather than on changing or alleviating symptoms. ACT techniques have been used to help people with CVD to avoid struggling with uncontrollable situations such as high blood sugar, amputation, and thoughts and feelings related to the disease. These techniques help people achieve a worthwhile life by embracing their inner experiences. ACT helped these patients understand what they were experiencing and not what they were feeling, thinking, or needing. This perspective allows people to act more flexibly when exposed to stimuli that trigger stress responses. Such psychological flexibility enables individuals to practice value-based self-compassion.^22,36^

With regard to emotion regulation in these patients, the results indicate that ACT improved the patients’ emotion regulation style. This result is in line with past studies in the ACT treatment field that have demonstrated that this therapy enhances emotion regulating abilities.^34,37^ Reappraisal often improves positive affect compared to a control condition without emotion regulation, which is consistent with data from correlational and experimental studies, suggesting that framing bad experiences in a more positive way may help people cope with them more appropriately and maintain a cheerful attitude, even when they experience excessive stress.^3^ In this context, recent research indicates that acceptance is one of the key components of ACT and can be an effective emotion regulation strategy. Diffusion and acceptance during ACT can be effective for improving disease-related emotional regulation styles.^3^ As a result, patients are helped and encouraged to accept their thoughts and feelings and to work on changing their thoughts and behaviors diligently. In this way, patients can perceive their intrusive thoughts as just thoughts and realize that their previous style of emotion regulation was ineffective. As a result, rather than reacting to thoughts, they learn to take steps toward what is important to them and what is consistent with their values.^38^ Thus, as Ruiz et al.^39^ suggest, ACT training can be used as an effective psychological intervention to improve emotion regulation.

This study's third finding shows that ACT could improve self-compassion in cardiovascular patients. This is consistent with studies that have shown that the therapeutic elements of this therapy and compassion-focused therapy are similar.^25^ Although self-compassion is not a stated objective of ACT, it does seem to be one of the pathways of change in ACT for CVD and chronic pain. Theoretically, ACT and self-compassion are related, and studies demonstrate that ACT fosters self-compassion. Compassion overlaps with ACT-promoted psychological processes and is one of the therapy-changing mechanisms underlying ACT for chronic pain conditions.^40^ In fact, self-compassion can be an important resource to help deal with the unique challenges of life caused by chronic physical illness.^40,41^ There is evidence that ACT is effective in increasing self-compassion. Several factors associated with this therapy may promote self-compassion, including unbiased or non-judgmental observation of one's own critical thinking, self-compassion through strengthening one's perspective, and self-acceptance.^40^ Therefore, in this therapy, self-compassion can be a key mediator through which the changes affect the intended outcome. This means that individuals who learn ACT may improve their well-being by adopting a compassionate attitude towards difficult experiences such as a serious illness, instead of trying to change them.^42^

This study had limitations that should be considered. The sample size was small. Due to the spread of the coronavirus, screening was conducted online and face-to-face interventions were conducted after the coronavirus epidemic subsided. Another limitation was the use of self-reporting tools. These tools have inevitable problems (evaluation error, not answering truthfully, etc.). Therefore, it is recommended that future studies be conducted using larger sample sizes and tools such as interviews.

## Conclusion

According to the findings of our study, patients who received treatment based on ACT experienced reductions in their psychological difficulties, while their capacity for self-compassion and emotion regulation increased. As a result, implementing this treatment and imparting its principles to patients can help them recover.
